# A survey on management of milk feeding, weaning and housing of conventional and organic dairy calves in Europe

**DOI:** 10.1186/s13028-025-00827-4

**Published:** 2025-09-30

**Authors:** Nina Dam Otten, Allison Welk, Margit Bak Jensen

**Affiliations:** 1 Department of Veterinary and Animal Sciences, Faculty of Health and Medical Sciences, University of Copenhagen, Grønnegårdsvej 8, Frederiksberg C, 1870 Denmark; 2 Department of Animal and Veterinary Sciences, Aarhus University, Blichers Allé 20, Tjele, DK-8830 Denmark; 3 Present Address: Department of Population Medicine, University of Guelph, Guelph, ON Canada

**Keywords:** Calf management, Dairy calves, Milk feeding, Questionnaire, Welfare

## Abstract

**Background:**

To safeguard dairy calf welfare, European legislative recommendations on milk feeding practices and minimum standards on housing of calves exist. However, studies providing a general overview of common practices on milk-fed calf rearing across European countries are sparse. The aim of this study was to provide an overview of current milk feeding and housing practices for dairy calves in conventional and organic herds across European countries. Forty-five respondents with extensive knowledge about dairy production and calf rearing from 25 countries and regions were invited to an online questionnaire regarding farm demographics, management of the newborn calves, milk feeding and housing practices of calves 1–4 weeks of age and 5–8 weeks of age, and weaning practices.

**Results:**

A total of 21 respondents from 15 countries and regions responded to the survey. The survey suggests that in conventional herds most calves spent a limited time with the dam after birth (≤ 12 h) with longer durations found in organic herds (> 2 days). Calves 1–4 weeks of age are reported to be commonly housed in individual pens and fed 6–8 L/day in two daily feedings. In most countries and regions, less than 25% of the herds are reported to be practicing *ad libitum* milk feeding. In most countries and regions, teat buckets or teat bars are reported to be used for milk feeding. In countries and regions where open buckets or troughs are more common, access to permanently mounted artificial dry teats (dummy teats) was typically provided. Calves 5–8 weeks of age are reported to be predominantly group housed and fed 8–10 L/day in two daily feedings with once a day milk feeding occurring more frequently in calves within this age group. Weaning was reported to be most frequently initiated between 8 and 10 weeks.

**Conclusions:**

Based on the respondents’ evaluations the survey suggests that there are discrepancies between recommendations based on research and the current practices regarding milk allowance and feeding frequency, and weaning. Legislative or industry regulations on timing of separation from the dam, milk type, or weaning age are primarily implemented for organic production systems in a smaller proportion of countries.

**Supplementary Information:**

The online version contains supplementary material available at 10.1186/s13028-025-00827-4.

## Background

Cultural and structural differences within the dairy sectors across European countries, and regions within countries, may influence calf-rearing methods [1, 2]. Over the past two decades there has been a shift in recommended dairy calf management practices [3] from facilitating concentrate intake through limited milk feeding (4 to 6 L/d) and early weaning (4 to 6 weeks) to now recommending that calves are fed higher milk allowances (10 to 12 L/d) and weaned at later ages (≥ 8 weeks). Dairy calves drink 12 to 16 L/d when offered milk *ad libitum* (e.g [4]). and several studies have found beneficial effects of *ad libitum* milk feeding, or high allowance milk, on growth and body composition of heifers [5–9]. Since calves are born monogastric they rely on milk as their primary source of energy and nutrients for the first three weeks of life, and calves under four weeks of age are unable to compensate for the energy loss from restricted milk allowances through sufficient increase in concentrate intake [5].

Insufficient milk supply (i.e. whole milk or milk replacer) has several negative welfare consequences for young calves not only in terms of growth [6, 10, 11], and immunosuppression [12], but also in terms of negative affective states such as frustration and hunger [13–15]. Dairy calves have a high motivation to suck in connection with milk intake, and failure to provide an outlet for this motivation results in a redirection of sucking to pen fixtures or other calves’ bodies [16]. In addition, feelings of hunger may intensify cross-sucking, a damaging behaviour where calves suck on other calves’ bodies [17, 18].

According to EU legislation calves should be fed at least twice a day with an “appropriate diet adapted to their age, weight, and behavioural and physiological needs, to promote good health and welfare” [19]. However, there is no legal requirement of this feed being milk and hence, and a qualitative interview study suggests that several different milk feeding strategies are used across EU member states including once daily milk feeding combined with additional feeding of concentrate and/or forage (e.g., hay, straw) [20].

In light of the increasing focus on the welfare of livestock, research has contributed to a better understanding of the welfare and economic consequences of challenges within calf rearing during the first weeks of life. Although many recent studies have investigated national conditions, studies providing a general overview of common practices on milk-fed calf rearing across countries and production systems in conventional and organic herds are sparse [21–23]. Summarising these different practices might facilitate further research or policymaking that potentially would govern better animal welfare for milk fed calves. The aim of the present survey is to gain insights to current calf rearing practices in European countries. Hence, the objectives of the study are, firstly, to provide an overview of milk feeding and rearing strategies for calves from birth to eight weeks of age by conducting a survey covering primarily replacement heifers and bull and heifer calves for fattening purposes until transfer to the fattening herds. Since organic farms are often additionally subject to further regulatory and/or industry standards, these potential differences for milk feeding and rearing practices are sought investigated as a secondary objective. To make the objectives feasible, a survey was sent to a convenience sample of dairy advisors, herd veterinarians, and researchers who had previously participated in a similar study on calving management [22] and usually visit a large number of dairy herds each year.

## Methods

The survey was conducted as an online country-level questionnaire on common calf management practices in conventional and organic herds and was conducted as a follow-up to the survey on management of cow and calf around the time of calving [22]; however, the present survey only covered European countries. Since the structural differences between conventional and organic farms can be of importance, respondents with a good knowledge of both production types were contacted. Respondents were identified by the authors (*n* = 32) based on their advisory activities or research within dairy calf management and their previous participation in the survey on calving management. Experts were selected based on three criteria: firstly, 12 experts who had participated in the previous study by Jensen & Tolstrup [22] on calving management were contacted. Secondly, another 12 experts were identified based on referrals within the authors professional networks; thirdly, 8 experts were identified either based on their contributions to the literature (Slovakia and Poland) or by searching profiles on the homepages of dairy advisory companies (3 experts in Norway, 2 experts in The Netherlands and 1 in Slovenia). In case any of the contacted experts did not see themselves qualified to answer the survey regading milk feeding practices, they were asked to refer to another expert with better expertise and more field experience on calf rearing practices. Respondents were selected as representatives for countries and for Germany and Portugal also for different geographical regions. A total of 45 respondents from 25 countries were contacted by email in October 2021, reminders were sent out in December 2021 and January 2022. The survey was closed, and data collection finalized by 5 March 2022 and all information was stored in anonymised form.

### Study population

Invitations to the survey were sent out to 45 respondents from 25 countries and responses were received from 21 respondents in 15 European countries: France (1), Germany (3), Ireland (1), Italy (1), Belgium (3), Austria (1), Slovakia (1), Portugal (1), Denmark (2), Sweden (1), Finland (1), the Netherlands (1), Norway (2), Latvia (1), and United Kingdom (1). No responses were received from Poland, Hungary, Romania, Bulgaria, Slovenia, Spain, Croatia, and Switzerland. No questionnaires were sent to Czech Republic, Greece, Luxembourg, Malta, and the Republic of Cyprus due to lack of contacts. Whenever possible, countries with large regional differences in dairy production settings (e.g., herd sizes or production type) were targeted by contacting multiple respondents. Hence, Germany was represented by three regions (North, East, and South), Portugal by three regions (North, South, and Azores), and Norway by two regions (East and West). Denmark was represented by two respondents to ensure coverage of both conventional and organic herds, as there are no distinct geographical distribution patterns of any specific production types.

### Questionnaire design

The survey was programmed using the online questionnaire software “SurveyXact by Rambøll” (Rambøll Management Consulting, Aarhus, Denmark) and was available in English only (Additional file 1). The aim was to identify common practices regarding calf management on dairy farms within the first eight weeks of life. Special emphasis was given to milk feeding and housing practices and regulations. After an initial introduction of the aim of the survey, definitions and scales for ranking were used. The questionnaire was divided into five parts. The first part contained general information of the participants background, annual farm visits, and general aspects of dairy production in the given country or region (e.g., herd size and housing). The following parts concerned colostrum management, milk feeding and housing of calves 1 to 4 weeks of age, milk feeding and housing of calves 5 to 8 weeks of age, and a final part regarding weaning. Comments and remarks were possible for each of the questions. Participants were asked to provide answers for three production types: conventional, certified organic and biodynamic (i.e. https://demeter.net) production. However, answers for the biodynamic herds were only supplied by Latvia and were excluded from results. The five sections covered 18, 7, 17, 15, and 8 closed questions, respectively. Respondents were either asked to give distributions (numerical), rank options, or choose the most appropriate option.

All respondents were asked for their consent to store the information in anonymised form. The questionnaire was pilot tested by one English-speaking and three native Danish researchers, and the full questionnaire is given the additional files[see Additional file 1]. Respondents from Germany were preselected to cover three different areas with different demographics for cattle herds, namely Schleswig-Holstein (North), Mecklenburg-Vorpommern (East), and Rhineland Palatinate/Bavaria/Hesse (South). Additionally, regional differences were specifically declared by experts from Portugal covering the North, the Southern regions of Alentejo and Ribatejo (South), and the Azores, and in Norway covering the regions Vestlandet Sør (West) and Østlandet Nord (East). In the present study Austria, Norway (East), and Germany (South) were considered as mountain regions.

## Results and discussion

The study aimed to provide an overview of the most common calf rearing practices regarding milk feeding and housing across the different dairy production systems in European countries. Results reported include descriptions of the common practices combined with the numeric answers when given. There were generally few comments added in the questionnaire. Comments were mainly on the low numbers of biodynamic herds in the given country or region, or on specific prohibitions (e.g., ban of milk replacer in organic herds in France, Norway, and Sweden) and experts lack of knowledge within a specific production type (5 experts stated limited knowledge in regards to organic herds). Albeit the housing of dairy cows and calves appear similar across countries, the management practices regarding milk feeding and weaning showed greater variation.

A response rate of 47% (21/45 respondents) was achieved for conventional herds. Respondents were veterinary practitioners (11), dairy cattle researchers (7), or affiliated with dairy advisory services (3). Questions regarding the organic herds showed a lower response rate of 36% (16/45 respondents). Answers from Belgium, Germany (East), and Ireland on organic production were not completed for questions other than the demographics. For Portugal (North) questions regarding organic systems were not applicable, since there are none in the specific regions according to the respondent. Hence, five respondents (Belgium, Germany (East), Ireland, Norway (East), Portugal (North)) did not answer for the organic production type leaving 76% of the replies answering questions regarding organic systems.

Initially, an inclusion criterion of ≥ 20 individual farm visits over the past 12 month was set; however, due to the covid-19 pandemic, many respondents were unable to meet this inclusion criterion. Three respondents indicated less than 10 individual farm visits. One respondent had only performed one herd visit during the given time period, while two respondents had performed > 50 visits in 4–5 herds. Under the given circumstances, the responses were still considered representative for the given country or region based on the authors’ knowledge on the respondents’ general experience.

Experts were chosen based on either the authors’ knowledge of their expertise within dairy calf rearing and dairy production settings through regular herd visits or by referral from national experts. Hence, experts’ replies should be regarded as main trends within the systems that these experts usually operate in and not as mean data. Although, the survey aimed to recruit experts with experience of a broad range of production types (i.e. conventional, organic, and biodynamic systems), only four experts gave estimates for the biodynamic systems but emphasised their sparse knowledge within these herds. Hence, the answers for the biodynamic herds were removed from the present study. Some experts also stressed the lack of data in the countries or regions where the number of organic herds are low. Organic herds are primarily found in the southern federal states in Germany and on the Azores in Portugal. Including experts from different regions seemed to be important in the present study, as there were evident differences between countries within housing and management of cows and calves in mountain regions. In the present study Portugal (Azores), Austria, Germany (South), and Norway were regarded as mountain regions. However, Slovakia also reported structural differences for dairy production settings in mountain regions. The country specific respondents answered that dairy herds in these regions were characterised as smaller holdings with typically less than 30 cows kept in tie stalls and seasonal pasture regardless of production type.

### Dairy herd characteristics

The distribution of organic herds was reported to vary across countries and regions from no herds in Portugal (North) to 22% in Austria (Table 1). Highest proportions of organic herds (> 10%) were reported for Austria, Sweden, Belgium, Denmark, Germany (South), and Portugal (South). Organic herds were reported to have numerically lower herd sizes across all countries except in the Nordic countries. Organic herds in Finland and Sweden were reported to have numerically higher herd sizes than conventional herds, while the two production types were reported to have approximately the same herd sizes in Denmark and Norway, which also was evident in The Netherlands. In general, the largest conventional herds (more than 200 cows on average) were reported for Portugal (South), Germany (East), United Kingdom, and Denmark, while the remaining countries and regions were reported to have 67–120 cows. Smallest herds (less than 50 cows) are found in Austria, Norway, and Portugal (Azores).

**Table 1:** Main characteristics of dairy herds in European countries and regions within countries of conventional (Conv) and organic (Org) herds

Country, region	Production type (%)		Herd size (N cows)		Most frequent housing of lactating cows		Access to summer pasture	Systems with summer pasture
	Conv	Org	Conv	Org	Conv	Org		
Austria	78	22	33	.	TS + P	LH-C	Yes	LH-C, TS
Belgium	90	10	120	.	LH-C	.	Yes	LH-DB
Denmark	85	15	253	130	LH-C	LH-C	Yes	LH-DB
Denmark	90	10	220	200	LH-C	LH-C	No	
Finland	97	3	51	67	LH-C	LH-C	No	
France	96	4	66	.	LH-C	.	Yes	LH-C, LH-DB
Germany, South	90	10	92	45	LH-C	LH-C	No	
Germany, East	95	5	300	.	P	.	Yes	NS
Germany, North	.	.	.	.	LH-C	LH-DB	No	
Ireland	95	5	84	.	LH + P	.	Yes	NS
Italy	96	4	70	50	LH-C	TS + P	Yes	NS
Latvia	90	10	50–300	50	LH-C	TS	Yes	NS
Norway, West	95	5	29.3	29.3	LH-C	LH-C	Yes	LH-C
Norway, East	97	3	28	32.1	TS	LH-C	Yes	LH-C, LH-DB
Portugal, North	100	0	70	0	LH-C	.	No	
Portugal, South	90	10	500	200	LH-C	LH-C	No	
Portugal, Azores	95	5	50	40	P (all year)	P (all year)	Yes	NS
Slovakia	92	8	110	.	TS + P	LH-DB	Yes	NS
Sweden	80	20	98	110	LH-C	LH-C	Yes	LHC, LH-DB, TS
The Netherlands	97	3	100	100	LH-C	LH-C	Yes	LH-C, LH-DB
United Kingdom	98	2	216	150	LH-C	LH-C	Yes	LH-C, LH-DB

Response option ‘Do not know’ or missing responses are indicated by ‘.’

LH(loose-housed), C(cubicles), DB (deep bedding), TS (tie stall), P (pasture), NS (NS)

Across all countries and regions loose housing with cubicles was the predominant housing type for cows in 71% of conventional (15/21 respondents) and 69% of organic herds (11/16 respondents). The combination of tie stalls and pasture was the most reported housing type in conventional herds in Austria and Slovakia, and organic herds in Italy. Tie stalls with no pasture access was the most reported housing type in conventional herds in Norway (East), while tie stalls with summer grazing was the most frequent housing type in organic herds in Latvia corresponding well to the relatively small average herd size of less than 50 cows per herd reported in the given countries. Access to summer pasture was reported to be a common practice by 37% of the respondents for cubicle systems and 47% of respondents for deep bedding systems. While the most frequently reported housing systems for dairy cows in the present study appear to be similar across countries and regions, dairy calf management practices showed large variation.

### Management of the newborn calf

The reported time of separation of cow and calf showed a similar pattern in conventional herds across countries and regions. The most reported time of separation was within the first 12 h after parturition (18/21 respondents). Only respondents from Portugal (Azores), Germany (North), and Denmark reported 12 to 24 h after birth as the most frequent time of separation. Time of separation varied among organic herds as 12/17 respondents (71%) reported that cows and calves were separated 12 to 24 h after birth while 5/16 respondents (35%) reported that cows and calves stay together for 2 to 7 days. Latvia reported longer time (> 8 days) before separation. The Netherlands and United Kingdom reported no difference in separation strategies between conventional and organic herds, where separation was reported to occur within the first 12 h after birth. The reported time of separation corresponded well to the reported methods of colostrum feeding (Table 2). Greatest difference reported where found between organic and conventional herds. Most calves were reported to be separated from the dam within 12 h after birth in conventional systems (reported by 85% respondents). In contrast, only 13% of respondents reported separation to occur within 12 h in organic systems with 40% reporting separation to occur at 12 to 24 h, and 40% reporting separation to occur between 2 and 7 days. National legislation on cow-calf separation was reported for both conventional and organic herds in Denmark, where calves are required to stay with their mother for at least 12 h, whereas Swedish legislation requires cows to be able to lick the calf dry before separation. Further codes of practice for organic production require calves to stay with their mothers for three days in Norway, for two days in Sweden, and for 24 h in Denmark. Cow calf separation is one of the welfare concerns of the public in relation to dairy production due to the perceived unnaturalness of the practice and there is an increasing public demand for extended cow-calf contact [24, 25]. In support of this, a systematic review finds that extended cow-calf contact has welfare benefits in terms promoting social behaviour and reducing abnormal oral behaviour [26]. The dairy industry has argued that separation before the cow-calf bond has formed (within days) is better because it leads to lower response to separation than separation after a few days, or weeks [27–29]. However, since little is known about the positive experiences of dam and calf while they are together, it is difficult to make the trade-off between these presumed positive and the negative effects of increased response to separation [16]. In addition, a systematic review concludes that extended cow-calf contact has shown inconsistent findings on increased risk of respiratory and enteric disease, as well as a lack of evidence describing absorption of immunoglobulins and mortality specifically [30]. However, it should be noted that in disease eradication programmes for paratuberculosis, immediate removal of the calf after birth has proven to be an effective intervention [31].

**Table 2:** Colostrum administration methods in relation to the time of separation of cow and calf. Percentages are given for the distributions within each production type

Time of separation	The most frequent method of feeding colostrum to the calf (%)					
	Conventional			Organic		
	Suckle from dam	Bottle feeding	Tube feeding	Suckle from dam	Bottle feeding	Tube feeding
Within 12 hours	0	85	5	0	13	0
12 to 24 hours	0	10	0	20	20	0
2 to 7 days	0	0	0	33	7	0
>8 days	0	0	0	0	7	0

Bottle-feeding was reported as the most common method for administering colostrum in conventional herds. In organic herds, 56% of responses reported suckling the dam as the most common method of colostrum feeding. Overall, bottle-feeding was reported as the most common colostrum feeding method in 7/16 countries or regions (44%). Tube feeding was only stated a frequent way of administering colostrum in conventional herds in Portugal (Azores). No differences were reported between production types in France, Italy, Latvia, Sweden, the Netherlands, and the United Kingdom. In general, time of separation and colostrum feeding methods differed more between production types than between countries or regions. Combining the information on colostrum feeding methods showed a numerically higher frequency of conventional herds (85%) practising separation within 12 h and bottle-feeding calves with colostrum, while there was a larger variation in the organic herds in regard to how long calves stay with the dam and receive their colostrum which is in line with previous European findings [32–35]. In North America, shorter intervals of cow-calf contacts were reported showing that most conventional herds separated calves from their dam before 6 h after parturition compared to 6–12 h in organic herds [36], while a more recent study reported 74.6% of herds separating cow and calf within 6 h after parturition [23]. Likewise, colostrum feeding was reported to be conducted primarily by teat feeding (bottle or bucket) [37, 38]. In addition, the increased focus on extended cow-calf contacted has led to an increase in European and Scandinavian farmers that allow calves to suckle their mothers for consecutive days [32–35].

### Milk feeding

Results indicate that the daily amount of milk fed to young calves (1 to 4 weeks of age) varies more between countries or regions than between production type (Table 3). The daily milk allowance most frequently reported within the first week of life in conventional herds was 6 to 8 L/day (10/21 respondents), followed by < 6 L/day (9/21 respondents). Most respondents reported an increase in milk allowance at 2 to 4 weeks of age. Germany (South) and Italy reported a constant daily milk allowance in both production types. Finland and Norway (West) reported higher milk allowances of 8 to 10 L/d across both production types. Milk allowances of 8 to 10 L/d were more common in organic herds than in conventional herds (milk allowance of 6 to 8 L/d); however, evaluation of the reported numbers should be made with caution due to the low number of respondents replying to this question for the organic herds. Previous studies report great variation in the daily milk allowance during the first weeks of life. From as low as 4.4 L/d reported in Spain [39], 5 L/d in Sweden [40], a peak volume of 5.5 L/d in Northern Ireland [41], 6 L/d in the Czech Republic [2] and 7 L/d in Norway [33]. In Austria almost 60% of farmers answered that calves were fed less than 12% of their body weight per day [35] compared to 25% feeding more than 12% of BW and 15% feeding *ad libitum*. In the present study, experts reported an increase in the daily milk allowance of the young calves over the first four weeks in some countries. This is more in line with recent studies performed in Western Germany by Hayer et al. [42] who reported the most common feeding practice of young calves to be more than 6 L/d (73.8% of farms). However, Mahendran et al. [43] reported lower milk allowance in the United Kingdom with 6 L/d being less frequently fed (21.8%) than ≤ 4 L/d (43.6%). Current recommendations state that dairy calves under four weeks of age should be fed approximately 20% of their body weight/d and studies have reported that *ad libitum* fed Holstein calves ingest on average 8.5 L/d of whole milk when 8 to 14 days old [44] and 9 to 11 L/d of whole milk when 3 to 6 weeks old [6]. On the other hand, calves offered limited amounts of milk (approx. 5 L/d) via a computer-controlled milk-feeder, pay twice as many unrewarded visits to the feeder as calves fed at least 8 L/d [14, 44, 45]. These high numbers of unrewarded visits are due to calves trying to get additional milk from the feeder and reflect hunger [45]. Other reported signs of hunger include frequent high-pitch vocalizations [13] and frequent butting of empty buckets after milk ingestion [46]. Research has shown that increasing the milk allowance from approximately 10–20% of body weight/d improves body weight gain and feed efficiency [3, 47] as well as reduced respiratory disease [48]. Additionally, Brown et al. [8] reported higher expression of udder parenchymal mass in calves fed a high energy, high protein diet compared to calves fed a moderate diet. This ‘metabolic programming’ has potential positive long-term effects in terms of improved reproductive and milk production within first lactation [49, 50]. In accordance, Welk et al. [51] concluded in their review, that higher milk allowances have positive effects on growth and positive emotional states of calves.

**Table 3:** Milk (replacer) feeding management in calves 1 to 4 weeks of age in conventional (Conv) and organic (Org) herds

Country	Volume of milk fed (L/d)								Number of daily milk feedings							
	1week		2week		3week		4week		1week		2week		3week		4week	
	Conv	Org	Conv	Org	Conv	Org	Conv	Org	Conv	Org	Conv	Org	Conv	Org	Conv	Org
Austria	< 6	6–8	< 6	8–10	6–8	8–10	6–8	8–10	3	> 3	2	2	2	2	2	2
Belgium	6–8	.	6–8	.	6–8	.	8–10	.	2	.	2	.	2	.	2	.
Denmark	6–8	6–8	6–8	6–8	6–8	6–8	6–8	6–8	2	2	2	2	2	2	2	2
Denmark	< 6	< 6	< 6	< 6	6–8	6–8	6–8	6–8	2	2	2	2	2	2	2	2
Finland	8–10	8–10	8–10	8–10	8–10	8–10	8–10	8–10	2	2	2	2	2	2	2	2
France	< 6	< 6	< 6	< 6	6–8	6–8	6–8	6–8	2	2	2	2	2	2	2	2
Germany, South	6–8	8–10	6–8	8–10	6–8	8–10	6–8	8–10	2	2	2	2	2	2	2	2
Germany, East	6–8	.	8–10	.	8–10	.	> 10	.	2	.	2	.	> 3	.	> 3	.
Germany, North	6–8	.	8–10	.	8–10	.	8–10	.	2	2	2	2	2	2	2	2
Ireland	< 6	.	6–8	.	6–8	.	6–8	.	2	.	2	.	2	.	2^a^	.
Italy	6–8	6–8	6–8	6–8	6–8	6–8	6–8	6–8	2	2	2	2	2	2	2	2
Latvia	6–8	8–10	8–10	8–10	8–10	> 10	8–10	> 10	2	> 3	2	> 3	2	> 3	2	3
Norway, West	8–10	8–10	8–10	8–10	8–10	8–10	8–10	8–10	3	> 3	3	3	3	3	3	3
Norway, East	6–8	8–10	6–8	8–10	.	.	< 6	6–8	3	.	3	.	.	.	.	.
Portugal, North	< 6	.	6–8	.	8–10	.	6–8	.	2	.	2	.	2	.	2	.
Portugal, South	6–8	6–8	6–8	8–10	6–8	8–10	6–8	8–10	2	3	> 3	> 3	> 3	> 3	> 3	> 3
Portugal, Azores	< 6	< 6	< 6	< 6	6–8	6–8	6–8	6–8	2	2	2	2	2	2	2^a^	2
Slovakia	6–8	.	8–10	.	8–10	.	8–10	.	2	.	2	.	2	.	2	.
Sweden	< 6	< 6	6–8	6–8	6–8	6–8	6–8	6–8	2	2	2	2	2	2	2	2
The Netherlands	< 6	< 6	6–8	6–8	6–8	6–8	6–8	6–8	2	2	2	2	2^a^	2^a^	2^a^	2^a^
United Kingdom	< 6	< 6	6–8	< 6	6–8	6–8	6–8	6–8	2	2	3	3	3	3	3^a^	3^a^

^a^ Once daily milk feeding may also occur

Response option ‘Do not know’ or missing responses are indicated by ‘.’

A general predominance of reports of two daily feedings within the first four weeks emerged in both production types across countries and regions (Table 3). However, Austria reported three daily feedings across both production types during the first week of age and Norway (East and West) and Portugal (South) during the first four weeks of age. The United Kingdom reported increases from two to three (or more) daily feedings at 2 weeks of age in both systems and Germany (East) at 3 weeks of age in conventional systems. Once a day milk feeding of young calves was reported as a common practice in a varying proportion of conventional herds across countries. Respondents reported 5% of herds using this practice in Belgium, Germany (South), and Denmark, 10% of herds in The Netherlands, 20% of the herds in France, and 25% of the herds in Ireland. In organic herds, respondents reported this practice in 2% of the organic herds in Denmark and 5% in France. In the Netherlands this practice was reported to commence in conventional herds as early as 3 weeks of age, while Ireland, the United Kingdom, and Portugal (Azores) started once a day milk feeding at 4 weeks of age in both organic and conventional herds. A potential welfare issue may be the introduction of once a day milk feeding in calves as early as at 3–4 weeks of age as was reported in the Netherlands, the United Kingdom, Ireland, and Portugal (Azores). Since the calves’ delayed ruminal development does not allow the calf to utilize solid feed efficiently until 8 weeks of age [52], reducing milk feeding frequency and milk allowance at an early age will likely reduce growth rates and increase signs of hunger. Previous studies have shown that calves with a reduced milk feeding frequency show hunger related behaviours such as spending more time on unrewarded visits to the milk feeder and on manipulating the teat [44, 53]. In order to compensate the lower energy intake, calves turn to alternate feed sources and are consuming larger amount of starter feed [54–56]. Reducing milk-feeding frequency may reduce labour costs, but only few studies have investigated the consequences of this practice. Some studies report that reducing milk feeding frequency among limit fed calves (10% of body weight/d) from twice to once daily did not significantly affect body weight gain (milk replacer: [57, 58]; whole milk: [59]), suggesting calves can ingest this low allowance in one single daily meal. However, one study with very young calves (3 to 8 days of age) fed milk replacer at 10% of body weight/day found that a reduced milk intake by approximately 50% on day three and by approximately 20% on day four when the milk feeding frequency was reduced from twice to once daily when calves were 3 days old. From 6 to 8 days of age, the milk intake was 10% higher in twice fed calves than once fed calves [60], suggesting that 1-week-old Holstein calves cannot ingest a milk allowance equivalent of 10% BW in one meal. Hubert et al. [61] reported an immediate increase in innate immune response when milk-feeding frequency was reduced from two to one in calves 3 week of age, suggesting they experience acute stress. Research on how once daily milk feeding affect milk intake and health when feeding higher milk allowances is still lacking. It is likely that once daily milk feeding will challenge the present recommendation of feeding a milk allowance of approx. 20% BW, and we encourage future research to investigate the welfare consequences of this practise in young and older milk fed dairy calves.

The daily milk allowance for calves 5 to 8 weeks was similar to young calves with milk levels of 8 to 10 L/d fed in Belgium, Finland, Germany (East), and Slovakia throughout the whole period in conventional herds (Table 4). Most responses indicated milk levels of 6 to 8 L/d in conventional herds (reported by 7/21 (33%) of the respondents) and organic herds (reported by 5/14 (36%) of the respondents). In conventional systems, a reduction in daily milk allowance to < 6 L/d at 6 weeks of age was reported in 24% of countries or regions (Austria, Norway (East), Portugal (North), Sweden, and United Kingdom). At 7 weeks of age, respondents in Germany (South), Norway (West), and Portugal (Azores) also reported reduced daily milk allowance, followed by Finland at 8 weeks. This pattern was not reported for the organic systems where the reduction seems to occur later and less frequent as 31% of respondents reported a reduction to < 6 L/d earliest at 6 weeks only in United Kingdom.

**Table 4:** Most common milk (replacer) feeding management in calves 5 to 8 weeks of age in conventional (Conv) and organic (Org) herds in 15 European countries

Country	Volume of milk fed (L/d)								Number of daily milk feedings							
	5week		6week		7week		8week		5week		6week		7week		8week	
	Conv	Org	Conv	Org	Conv	Org	Conv	Org	Conv	Org	Conv	Org	Conv	Org	Conv	Org
Austria	6–8	8–10	< 6	6–8	< 6	6–8	< 6	6–8	2	2	1	2	1	2	1	2
Belgium	8–10	.	8–10	.	8–10	.	8–10	.	2	.	2	.	2	.	2	.
Denmark	6–8	6–8	6–8	6–8	6–8	6–8	6–8	6–8	2	2	2	2	2	2	1	2
Denmark	6–8	6–8	6–8	6–8	6–8	6–8	6–8	6–8	2	2	2	2	2	2	2	2
Finland	8–10	8–10	8–10	8–10	8–10	8–10	< 6	8–10	2	2	2	2	2	2	1	2
France	6–8	6–8	6–8	6–8	6–8	6–8	6–8	6–8	2	2	2	2	2	2	2	2
Germany, South	6–8	8–10	6–8	8–10	< 6	6–8	< 6	< 6	2	2	2	2	2	2	1	1
Germany, East	8–10	.	>10	.	8–10	.	8–10	.	> 3	.	> 3	.	> 3	.	3	.
Germany, North	6–8	.	6–8	.	6–8	.	6–8	.	2	2	2	2	2	2	2	2
Ireland	6–8	.	6–8	.	6–8	.	< 6	.	2	.	2	.	2	.	2	.
Italy	6–8	6–8	6–8	6–8	6–8	6–8	6–8	6–8	2	2	2	2	2	2	2	2
Latvia	8–10	>10	6–8	8–10	6–8	8–10	6–8	8–10	2	3	2	3	2	3	2	2
Norway, West	6–8	6–8	6–8	6–8	< 6	< 6	< 6	< 6	2	2	2	2	2	2	1	1
Norway, East	6–8	.	< 6	.	< 6	.	< 6	.	2	.	2	.	2	.	2	.
Portugal, North	6–8	.	< 6	.	< 6	.	< 6	.	2	.	2	.	2	.	2	.
Portugal, South	8–10	8–10	6–8	8–10	6–8	6–8	< 6	6–8	> 3	> 3	> 3	> 3	2	3	2	3
Portugal, Azores	8–10	8–10	6–8	6–8	< 6	< 6	< 6	< 6	2	2	2	2	2	2	2	2
Slovakia	8–10	.	8–10	.	8–10	.	8–10	.	2	.	2	.	2	.	2	.
Sweden	6–8	6–8	< 6	6–8	< 6	6–8	< 6	< 6	2	2	2	2	2	2	2	2
The Netherlands	6–8	6–8	6–8	6–8	6–8	6–8	6–8	6–8	2	.	2	.	2	.	2	.
United Kingdom	6–8	6–8	< 6	< 6	< 6	< 6	< 6	< 6	2	2	2	2	2	2	1	1

Response option ‘Do not know’ or missing responses are indicated by ‘.’

Twice a day milk feedings of calves 5 to 8 weeks was most common in both conventional (86% of responses) and organic (86% of responses) systems. Once a day milk feeding was reported by 6/21 of the respondents (29%) for conventional systems and 3/14 of the respondents (21%) for organic systems, and appear to be related to the reduction in daily milk allowance. However, 13 respondents for conventional herds and five respondents for organic herds reported that farmers do implement once a day milk feeding most commonly around 6 to 8 weeks, which appeared to be related to the reduction of daily milk allowance around weaning. Once a day milk feeding was more frequent in older calves (5–8 weeks of age) ranging from 5 to 80% of conventional herds across countries and regions and 5 to 25% of the organic herds (Table 5). The mean (± SD) age of calves, when starting the once daily milk feeding, was 6 weeks (± 1.86 week) in conventional and 7 weeks (± 2.1 weeks) in organic herds. Lowest age for this practice was found in The Netherlands, United Kingdom, and Ireland (3 to 4 weeks) (Table 5). According to respondents, *ad libitum* feeding of milk most frequently occurs to a minor extent, meaning that less than 25% of herds use this practice in both conventional (73% of responses) and organic herds (70% of responses). Milk replacer is the most common milk type used for this age groups in conventional systems (reported by 13/21 (62%) of the respondents), while it is more frequently used in combination with whole and/or waste milk in organic systems (reported by 5/17 (29%) of the respondents). Thus, whole milk appears to play a bigger role in the organic systems across all countries and regions.

**Table 5:** Once daily milk feeding and the onset of this practice (Age in weeks) and occurrence of ad libitum feeding of dairy calves within weeks 5 to 8 in in conventional (Conv) and organic (Org) herds

Country		One daily feeding (%)			Age in weeks			Ad libitum feeding	
		Conv	Org		Conv	Org		Conv	Org
Austria		70	0		6	8		.	.
Belgium		20	0		8	.		Minor extent (< 25% of farms)	.
Denmark		25	5		8	10		Minor extent (< 25% of farms)	Minor extent (< 25% of farms)
Finland		.	.		.	.		Moderate extent (> 50% of farms)	Great extent (> 75% of farms)
France		30	5		6	8		Minor extent (< 25% of farms)	Minor extent (< 25% of farms)
Germany, South		25	25		7	7		Minor extent (< 25% of farms)	Minor extent (< 25% of farms)
Germany; East		.	.		.	.		Minor extent (< 25% of farms)	.
Germany, North		80	0		.	.		Moderate extent (> 50% of farms)	.
Ireland		50	0		4	4		Moderate extent (> 50% of farms)	.
Italy		0	0		.	.		Minor extent (< 25% of farms)	Minor extent (< 25% of farms)
Latvia		50	0		5	.		Minor extent (< 25% of farms)	Moderate extent (> 50% of farms)
Norway, West		5	5		8	8		Minor extent (< 25% of farms)	Minor extent (< 25% of farms)
Norway, East		0	0		7	.		Minor extent (< 25% of farms)	.
Portugal, North		5	0		9	.		Minor extent (< 25% of farms)	.
Portugal, South		.	.		.	.		Great extent (> 75% of farms)	Great extent (> 75% of farms)
Portugal, Azores		10	0		4	.		.	.
Slovakia		.	.		.	.		Minor extent (< 25% of farms)	.
Sweden		.	.		.	.		Minor extent (< 25% of farms)	Minor extent (< 25% of farms)
The Netherlands		10	0		3	.		Minor extent (< 25% of farms)	.
United Kingdom		1	1		4	4		Minor extent (< 25% of farms)	Minor extent (< 25% of farms)

Response option ‘Do not know’ or missing responses are indicated by ‘.’

The reported type of milk fed within the first 4 weeks of life differed more between countries and regions than production types. The most frequent type of milk, which was reported to be fed to young calves (1–4 weeks of age), was whole milk in combination with milk replacer in conventional herds (48%) and whole milk in organic herds (50%). Milk replacer was the most common milk type used in conventional herds in Belgium, Finland, Portugal (North), Slovakia, Sweden, the Netherlands, and the United Kingdom. Only the Netherlands reported milk replacer as the most common type of milk fed to young calves (1–4 weeks of age) in organic herds. Respondents from France, Norway, and Sweden indicated that the use of milk replacer in organic herds is not allowed by codes of practice in their respective countries. Waste milk was used less frequently than whole milk and was reported to predominantly derive from cows with elevated somatic cell count in conventional herds (14/20 (70%) responses) and organic herds (13/14 (93%)). One third of respondents (Belgium, Ireland, Latvia, Germany (South), Germany (East), Germany (South)) reported that waste milk was derived from cows treated with antibiotics in conventional herds. Germany (North) reported waste milk derived from cows treated with antibiotics was used in both conventional and organic herds. However, there is a lack of knowledge on how different milk types might affect the health of calves.

The milk feeding method used for young calves (1–4 weeks of age) was reported to differ more between countries and regions than between production types (Table 6). The most frequent milk feeding method reported was by teat bucket or teat bar from 15/21 of the respondents (71%) regarding conventional herds and 11/16 of the respondents (69%) regarding organic herds. Only Denmark reported open troughs or buckets as the most frequent milk feeding method used in both systems, and only the United Kingdom reported automated milk feeders as the most frequent milk feeding method used in both systems. In organic herds in Germany (South), Latvia, and Slovakia, calves were reported to be predominantly kept with their mothers during the first week of life, while this also was reported as the second most frequent scenario in Belgium, Finland, Norway, and Portugal (Azores).

**Table 6:** Management practices regarding milk (replacer) feeding and provision of artificial teats for dairy calves in conventional (Conv) and organic (Org) herds

Country	Most frequent way of feeding milk to dairy calves					Access to artificial teat	
	Week 1 to 4		Week 5 to 8				
	Conv	Org	Conv	Org		Conv	Org
Austria	Teat bucket/bar	Teat bucket/bar	Teat bucket/bar	Teat bucket/bar		Feeding	Feeding
Belgium	Teat bucket/bar	Teat bucket/bar	Teat bucket/bar	.		Permanent	.
Denmark	Open trough/bucket	Teat bucket/bar	Open trough/bucket	Open trough/bucket		Permanent	Permanent
Denmark	Open trough/bucket	Teat bucket/bar	Open trough/bucket	Open trough/bucket		Permanent	Permanent
Finland	Teat bucket/bar	Teat bucket/bar	Teat bucket/bar	Teat bucket/bar		Permanent	Permanent
France	Teat bucket/bar	.	Open trough/bucket	Open trough/bucket		Feeding	Permanent
Germany, South	Teat bucket/bar	Mother/nurse	Automatic milk feeding	Teat bucket/bar		Permanent	Feeding
Germany; East	Teat bucket/bar	.	Automatic milk feeding	.		Permanent	Feeding
Germany, North	Teat bucket/bar	.	Teat bucket/bar	Teat bucket/bar		Feeding	.
Ireland	Teat bucket/bar	.	Teat bucket/bar	.		Feeding	Feeding
Italy	Teat bucket/bar	Teat bucket/bar	Teat bucket/bar	Teat bucket/bar		Feeding	.
Latvia	Teat bucket/bar	Mother/nurse	Automatic milk feeding	Open trough/bucket		Feeding	Feeding
Norway, West	Teat bucket/bar	Teat bucket/bar	Teat bucket/bar	Teat bucket/bar		Feeding	Feeding
Norway, East	Teat bucket/bar	Teat bucket/bar	Teat bucket/bar	.		Feeding	Feeding
Portugal, North	Open trough/bucket	.	Open trough/bucket	.		Feeding	.
Portugal, South	Teat bucket/bar	Teat bucket/bar	Automatic milk feeding	Automatic milk feeding		Feeding	.
Portugal, Azores	Open trough/bucket	Teat bucket/bar	Teat bucket/bar	Teat bucket/bar		No access	.
Slovakia	Open trough/bucket	Mother/nurse	Open trough/bucket	Mother/nurse		Feeding	Feeding
Sweden	Teat bucket/bar	Teat bucket/bar	Open trough/bucket	Teat bucket/bar		Feeding	.
The Netherlands	Teat bucket/bar	Teat bucket/bar	Automatic milk feeding	Automatic milk feeding		Feeding	Feeding
United Kingdom	Automatic milk feeding	Automatic milk feeding	Automatic milk feeding	Automatic milk feeding		Permanent	Permanent

Response option ‘Do not know’ or missing responses are indicated by ‘.’

Access to artificial teats (e.g., milk teat in automatic milk feeder or teat bucket/bar) at milk feeding was reported as being provided by 14/21 of the respondents (67%) regarding conventional systems and 10/14 of the respondents (71%) regarding organic systems. Permanent access to mounted artificial teats (i.e. latex rubber teats, also termed ‘dummy teats’) was the case in 6/21 (29%) and 4/14 (29%) responses for conventional and organic systems, respectively (Table 6). Portugal (Azores) responded that calves in conventional herds do not have access to an artificial teat. Legal requirements for providing calves with an outlet to suck in connection with milk intake (i.e., feeding milk via a teat, giving access to dummy teats, or dam-rearing) were reported in Denmark covering both conventional and organic systems. Organic calves are ensured this access by legislation in Austria and Sweden, while this issue is covered by codes of practice in Norway. The Danish national legal requirements for feeding via a teat, or providing a dummy teat, was enforced aiming to meet the calves’ inherent behavioural need to suck in connection with milk ingestion, while this is ensured by codes of practice only for organic herds in Austria and Sweden. Nonetheless, experts from both the latter countries stated feeding via a milk teat as the most common practice. Norway, which is not an EU member state, has legal requirements regarding housing whilst access to dummy teats are covered by codes of practice for both conventional and organic herds. All EU member states are obliged to comply with the EU Directive 2008/119/EC, setting out the minimum standards for the protection of calves. According to the respondents asked in the present study, the majority of herds are providing an opportunity for calves to suck in connection with milk feeding, while the remaining respondents indicated permanent access to artificial or dummy teats, except for Portugal (Azores) where milk feeding via artificial teats or access to dummy teats are not commonly used. Although the use of dummy teats in combination with feeding milk in open buckets has been recommended to prevent cross-sucking [62], research has shown that dummy teats are more attractive when dipped in milk [18]. They are likely to be used more if close to the milk source, as dummy teats placed away from the teat bucket in individual hutches did not affect the time calves spent sucking on pen fixtures [36]. A recent study found that bucket-fed calves with access to a Braden bottle (a bottle with a dry teat filled with grain) spent less time cross-sucking than bucket-fed calves with no access to a Braden bottle. However, access to a Braden bottle did not reduce time spent on cross-sucking in bucket-fed calves compared to teat-fed calves [63]. Therefore, feeding the milk via a teat by using a teat bucket, teat bar or automatic milk feeder is more likely to satisfy the calves need to suck and to prevent cross-sucking than offering dry teats after feeding the milk in open buckets or troughs. We encourage research to investigate the effect of feeding milk via a teat compared to offering a dry teat, especially under group housing conditions, to support future legislation and recommendations.

### Housing

Housing type differed between both systems and countries or regions (Table 7). Individual housing was the predominant housing type reported for young calves with 86% in conventional and 60% in organic herds. In organic herds social housing types were reported more common, e.g. pair housing in Denmark and housing with dam/nurse cow in Italy, Latvia, and Portugal (South). After separation from the dam, pre-weaned calves were most commonly housed in single pens or huts, which is in line with epidemiological findings in Norway [33], Sweden [35], Austria [35], and the Czech Republic [2]. Organic herds in Denmark [64] and herds enrolled in an official animal welfare-labelling scheme (BEK nr 537 28/05/2024) are required to pair house calves immediately after separation from the dam). Group housing, or social housing, was also the most reported housing type for older calves in both conventional (reported by 17/21 respondents (81%)) and organic systems (reported by 14/16 respondents (87.5%)). In Germany (East), Italy, and Slovakia individual housing was the most reported housing type in conventional systems, while pair housing of calves was most frequently used in conventional systems in Austria and organic systems in Italy. In Slovakia, older calves were reported to be housed with the dam or nurse cow until 8 weeks.

**Table 7:** Typical management practices regarding housing across 15 European countries in conventional (Conv) and organic (Org) herds

Country	Most frequent way of housing dairy calves					
	Week 1 to 4			Week 4 to 8		
	Conv	Org		Conv	Org	
Austria	Individual	Individual		Pair	Group	
Belgium	Individual	.		Group	.	
Denmark	Pair	Pair		Group	Group	
Denmark	Individual	Pair		Group	Group	
Finland	Individual	Group		Group	Group	
France	Individual	Individual		Group	Group	
Germany, South	Individual	Individual		Group	Group	
Germany; East	Individual	.		Individual	.	
Germany, North	Individual	Individual		Group	Group	
Ireland	Group	.		Group	.	
Italy	Individual	Cow/nurse		Individual	Pair	
Latvia	Individual	Cow/nurse		Group	Group	
Norway, West	Individual	Individual		Group	Group	
Norway, East	Group	.		Group	.	
Portugal, North	Individual	.		Group	.	
Portugal, South	Individual	Cow/nurse		Group	Group	
Portugal, Azores	Individual	Individual		Group	Group	
Slovakia	Individual	.		Individual	Cow/nurse	
Sweden	Individual	Individual		Group	Group	
The Netherlands	Individual	Individual		Group	Group	
United Kingdom	Individual	Individual		Group	Group	

Response option ‘Do not know’ or missing responses are indicated by ‘.’

Focus on social housing of pre-weaned calves is gaining in popularity as market driven welfare schemes are increasing. Social housing has been reported to have beneficial effects on calf welfare by enabling social bonding and affiliative behaviour [65] lowered fear levels [66, 67] and increased feed intake [68]. Furthermore, Svensson et al. [69] found a similar risk of respiratory disease among individually housed calves and pair-housed calves, while the risk depended on group size with a higher risk in larger groups compared to smaller groups. In line with these findings, a recent report on the welfare in calves recommends that calves be housed in pairs or small groups from the age of 1 week [1].

### Weaning

The most common criteria to initiate weaning was age (reported by 95% of experts in conventional and 100% of experts in organic systems). Only in Portugal (South), concentrate intake was reported as the most frequent criteria used to wean calves in conventional systems but not organic. The most common weaning age occurred between 8 and 10 weeks (reported by 12/21 respondents (57%) in conventional and 4/15 respondents (27%) in organic systems). The earliest weaning age was at 6 to 8 weeks in conventional herds in Latvia, Norway (East and West), and the United Kingdom. Weaning after 10 weeks was the most common weaning age in organic systems (11/15 respondents (73%)). Only 5/21 respondents (24%) reported weaning after 10 weeks in conventional systems. Weaning over seven days was the most common weaning duration (reported by 13/21 respondents in conventional and 10/14 respondents in organic systems). Abrupt weaning was not reported as a common practice in any of the involved countries or regions; however, in Latvia weaning was reported to be commonly performed over less than five days.

These results suggest that there is a great variation in the onset of weaning across countries and regions and between production systems, which is consistent with previous studies indicating an average weaning age of 8 to 10 weeks in England and Wales [70], the Czech Republic [2], and Sweden [40]. Later weaning at more than 10 weeks has been reported in France [71], Spain [39], Germany [42], and Austria [35]. Weaning age has shown to have a significant effect on weaning stress in dairy calves in terms of increased abnormal behaviour, vocalizations, and lying behaviour when weaned at 6 weeks compared to 8 weeks [72]. Hence, significant welfare improvements might be achieved by implementing weaning later than the commonly used eight weeks and by establishing minimum weaning ages within the legislation, as is the case for weaning of piglets given by the EU directive 2008/120/EEC and by industry codices for calves in organic herds in some of the European countries.

### Differences between conventional and organic herds

The present survey indicated differences in calf rearing practices between organic and conventional herds within the areas of cow-calf contact, onset of milk reduction and weaning, once daily feeding, access to artificial teats and social housing of calves. The legal framework for organic production for EU member states are given by the Commission Regulation 2018/848(EC), however, only few of these areas identified here are covered by legal regulations. Milk feeding for at least 90 days, social housing of calves from 1 week of age and requirement of natural feed are legal requirements specifically for organic dairy calves. On the other hand, all dairy calves both conventional and organic are also legally covered by the EU Directive 2008/119/EC, in which the provision of calf-calf contact (“see/touch”), space allowance, access to fibrous feed and twice a day feeding are contained. The sparse additional legal requirements for organic production do not really harmonise with the organic concept of “naturalness”, and hence most organic federations have implemented further industry codices to encompass more natural behavioural needs like cow-calf contact and suckling behaviour. In many countries stricter national rules as well as a variety of quality assurance programmes have also set out more detailed welfare rules to ensure better rearing conditions and to meet the market driven welfare requirements. Thus, solely relying on organic labelling does not seem to warrant significantly better animal welfare [73]. Nonetheless, the present survey showed a higher occurrence of practices with a documented welfare benefit.

### Limitations

As the present data is based on an expert survey and as the replies were not investigated or validated further, data should be interpreted with caution. Since the questionnaire was only available in English, the translation and comprehension of the questionnaire by non-native speakers were not pre-tested, as suggested by Bujang et al. (2022) [74] potentially introducing a confounding effect. An important limitation of the present study was the low number of experts for each country or region. The present study only used one expert per country/region, which could introduce bias as experts might only visit certain types of farms e.g. larger scale operations and/or more progressive herds. For instance, it is a concern, that the respondent’s perception of automated milk feeders with three daily feedings being the most common feeding method in the United Kingdom was not in line with findings by Mahendran et al. (2022), who reported manual feeding in individual buckets, or teat feeders, as the most common methods in the United Kingdom. Whereas Mahendran et al. (2022), as well as others, relied on farmers reporting their practices to generate evidence on a national level; the present study presents the experience of experts and generates an overview of the most common practices in 15 European countries. Including more experts per country and region would help solidify the results; however, including farmers across all these countries would have demanded an enormous effort and vast resources.

## Conclusions

The current study presents an overview of the most common calf rearing practices in conventional and organic herds regarding milk feeding and housing across European countries. Due to geographic differences across countries and regions, calf-rearing practices show a great variation. In general, the reported milk allowances and feeding frequencies are low compared to current evidence on voluntary milk ingestion. Younger calves 1 to 4 weeks of age are especially at risk of compromised welfare in particular due to the lack of compliance with current research and management recommendations in terms of milk allowances, the number of daily feedings, and weaning practices. The industry codices and regulations for organic dairy systems seem more closely aligned with current recommendations, aiming to enhance animal welfare. This was evident in practices such as extended cow-calf contact, social housing, and the later onset of weaning. Implementing research findings on general practices in regards to these issues, at least in recommendations or even in a legislative context, may further increase and ensure calf welfare.

## Electronic supplementary material

Below is the link to the electronic supplementary material.

Supplementary Material 1

## Data Availability

The datasets used and/or analysed during the current study are available from the corresponding author on reasonable request.

## Declarations

### Prior publication

Data have not been published previously.
