# Supplementary material for: A survey on management of milk feeding, weaning and housing of conventional and organic dairy calves in Europe
Source: Acta Vet Scand. 2025 Sep 30;67:45. doi: 10.1186/s13028-025-00827-4 (PMC12482143; doi:10.1186/s13028-025-00827-4)
Supplement: Supplementary file 1 — Supplementary Material 1 [file 13028_2025_827_MOESM1_ESM.pdf]

Background and aim of survey (as described in e-mail)

Milk feeding recommendations have focused on the total amount fed per day in order to optimize calf growth and health. However, the calf welfare should also encompass behavioural aspects, in which the methods and frequency of milk feedings also play an important role. Legislative requirements and codes of practice for calf feeding, housing and management for different production types, namely organic herds, may differ between countries, while structural differences between regions of countries also may differ. Therefore, the present survey intends to provide an overview of the most common milk feeding regimes of dairy calves in a number of European countries The survey is funded by the Danish Centre for Animal Welfare and carried out by Nina Dam Otten, University of Copenhagen and Margit Bak Jensen, Aarhus University.

As a dairy researcher, or advisor, you hold valuable knowledge of dairy calf feeding strategies and management of dairy calves in your country, or region. Please, share this knowledge by completing this questionnaire. It should take no more than 20-30 minutes. Before storage and analysis, the data will be anonymized.

If there are different production systems (e.g. smallholder vs. large-scale production, conventional vs. organic production) in the country, or the region, that your reply concerns, please also state this and describe the production system your reply concerns. In the questionnaire, you will asked to reply concerning a country, production systems within a country, or a region, or a production system within a region.

Once we have the results of the survey, we will share them with you either on the two universities webpages, or as a short communication in a scientific journal.

Introduction to scale

For some questions, you will be asked to categorize options from the most frequent to the least frequent. The score 1 is given the option that occurs most frequent and the highest score is given the least frequent option. Reply to the best of your knowledge.

GENERAL INFORMATION

Country of residence:___________________________

Institution/employer

____

Profession - which is/was predominant profession during the past 12 months?

(1) ❑ Animal Husbandry advisor, dairy cattle

(10) ❑ Veterinarian, dairy cattle

(11) ❑ Researcher, dairy cattle

Number of dairy farms visited within the past 12 months?

(1) ❑ Under 10

(4) ❑ 10 to 20

(5) ❑ 20 to 30

(6) ❑ 30 to 40

(7) ❑ 40 to 50

(8) ❑ Over 50

(9) ❑ Do not know

Are there regional differences in your country of residence concerning milk feeding (e.g. geographical differences, or differences between regions, provinces or states)?

(1) ❑ Yes, this reply concerns a specific region (please specify below)

(2) ❑ No, this reply concerns my country of residence

(3) ❑ Do not know

Please answer the following question for your country of residence:

Are there very different production systems (e.g. smallholder vs. large-scale production, or conventional vs. organic production) in your country of residence?

(1) ❑ Yes

(2) ❑ No

(3) ❑ Do not know

What are the estimated percentages (your best guess) of production systems among dairy farms in your country (or region)?

Conventional herds _____

Organic herds _____

Biodynamic herds _____

Do not know (if you choose this answer, please set at 100 %) _____

What is the average number of dairy cows per farm?

If you do not know, please set the value at 100 for the "Do not know" option

Conventional herds _____

Organic herds _____

Biodynamic herds _____

Do not know _____

What is the most frequent type of housing for lactating cows?

	Loose housing system with cubicles (free-stalls)	Loose housing system with deep bedding	Tie stall	Pasture (all year)	Other (please describe below)	Do not know
Conventional farms	(8) ❑	(13) ❑	(9) ❑	(10) ❑	(11) ❑ ____	(12) ❑
Organic farms	(8) ❑	(13) ❑	(9) ❑	(10) ❑	(11) ❑ ____	(12) ❑
Biodynamic and others	(8) ❑	(13) ❑	(9) ❑	(10) ❑	(11) ❑ ____	(12) ❑

Are lactating cows in conventional farms typically on summer pastures in the following housing systems?

	Yes	No	Do not know
Loose housing system with cubicles (free-stalls)	(1) ❑	(2) ❑	(3) ❑
Loose housing system with deep bedding	(1) ❑	(2) ❑	(3) ❑
Other (if not relevant, choose "Do not know")	(1) ❑	(2) ❑	(3) ❑

Are lactating cows in organic and biodynamic farms typically on summer pastures in the following housing systems?

	Yes	No	Do not know
Loose housing system with cubicles (free-stalls)	(1) ❑	(2) ❑	(3) ❑

	Yes	No	Do not know
Loose housing system with deep bedding	(1) ❑	(2) ❑	(3) ❑
Other (if not relevant, choose "Do not know")	(1) ❑	(2) ❑	(3) ❑

When are cow and calf most commonly separated?

	Within 12 hours after birth	12 to 24 hours	2 to 7 days	> 8 days	Do not know
Conventional farms	(1) ❑	(2) ❑	(3) ❑	(4) ❑	(5) ❑
Organic farms	(1) ❑	(2) ❑	(3) ❑	(4) ❑	(5) ❑
Biodynamic and others	(1) ❑	(2) ❑	(3) ❑	(4) ❑	(5) ❑

CALF MANAGEMENT

How many people are attending the milk-fed calves on a daily basis?

	1 person	1-2 different persons	2-4 different persons	> 4 different persons	Do not know
Conventional	(1) ❑	(2) ❑	(3) ❑	(4) ❑	(5) ❑
Organic	(1) ❑	(2) ❑	(3) ❑	(4) ❑	(5) ❑
Biodynamic and others	(1) ❑	(2) ❑	(3) ❑	(4) ❑	(5) ❑

Who is responsible for setting up the management routines regarding calves?

	Farm manager/Owner	Calf manager	Non-specific calf feeder	Do not know
Conventional farms	(1) ❑	(2) ❑	(3) ❑	(5) ❑
Organic farms	(1) ❑	(2) ❑	(3) ❑	(5) ❑
Biodynamic and others	(1) ❑	(2) ❑	(3) ❑	(5) ❑

Who is the primary caretaker of the milk-fed calves?

	Calf or farm manager/Owner	Specific calf feeder	No specific person (alternating staff members)	Do not know
Conventional farms	(1) ❑	(2) ❑	(3) ❑	(5) ❑
Organic farms	(1) ❑	(2) ❑	(3) ❑	(5) ❑
Biodynamic and others	(1) ❑	(2) ❑	(3) ❑	(5) ❑

What is the predominant sex of the primary caretakers?

	Female	Male	Do not know
Conventional farms	(1) ❑	(2) ❑	(8) ❑
Organic farms	(1) ❑	(2) ❑	(8) ❑
Biodynamic and others	(1) ❑	(2) ❑	(8) ❑

What is the predominant background of the primary caretakers?

	No formal education	Vocational training	Agricultural training	Graduate training	Do not know
Conventional farms	(6) ❑	(3) ❑	(5) ❑	(7) ❑	(8) ❑

	No formal education	Vocational training	Agricultural training	Graduate training	Do not know
Organic farms	(6) ❑	(3) ❑	(5) ❑	(7) ❑	(8) ❑
Biodynamic and others	(6) ❑	(3) ❑	(5) ❑	(7) ❑	(8) ❑

How do farms manage their milk-feeding routines?

	Systematic routines	Partly systematic routines	No strict routines	Do not know
Conventional farms	(9) ❑	(10) ❑	(11) ❑	(8) ❑
Organic farms	(9) ❑	(10) ❑	(11) ❑	(8) ❑
Biodynamic and others	(9) ❑	(10) ❑	(11) ❑	(8) ❑

CALVES – COLOSTRUM FEEDING PERIOD

What is the main method for calves to obtain colostrum?

	Suckle from dam	Bottle feeding	Tube feeding	Do not know
Conventional farms	(1) ❑	(6) ❑	(3) ❑	(5) ❑
Organic farms	(1) ❑	(6) ❑	(3) ❑	(5) ❑
Biodynamic and others	(1) ❑	(6) ❑	(3) ❑	(5) ❑

Do farms make use of colostrum banks?

	Yes, to a great extend (> 75 % of farms)	Yes, to a moderate extend (>50 % of farms)	Yes, to a minor extend (< 25 % of farms)	Do not know
Conventional farms	(1) ❑	(6) ❑	(3) ❑	(5) ❑

	Yes, to a great extend (> 75 % of farms)	Yes, to a moderate extend (>50 % of farms)	Yes, to a minor extend (< 25 % of farms)	Do not know
Organic farms	(1) ❑	(6) ❑	(3) ❑	(5) ❑
Biodynamic and others	(1) ❑	(6) ❑	(3) ❑	(5) ❑

Is the colostrum quality checked prior to feeding or freezing?

	Yes, to a great extend (> 75 % of farms)	Yes, to a moderate extend (>50 % of farms)	Yes, to a minor extend (< 25 % of farms)	Do not know
Conventional farms	(1) ❑	(6) ❑	(3) ❑	(5) ❑
Organic farms	(1) ❑	(6) ❑	(3) ❑	(5) ❑
Biodynamic and others	(1) ❑	(6) ❑	(3) ❑	(5) ❑

Is the colostrum pasteurized?

	Yes, to a great extend (> 75 % of farms)	Yes, to a moderate extend (>50 % of farms)	Yes, to a minor extend (< 25 % of farms)	Do not know
Conventional farms	(1) ❑	(6) ❑	(3) ❑	(5) ❑
Organic farms	(1) ❑	(6) ❑	(3) ❑	(5) ❑
Biodynamic and others	(1) ❑	(6) ❑	(3) ❑	(5) ❑

How many colostrum feedings do calves in conventional farms receive from the:

	1 feeding/day	2 feedings/day	3 feedings/day	>3 feedings/day	Do not know
First milking of the dam	(8) ❑	(13) ❑	(14) ❑	(15) ❑	(5) ❑

	1 feeding/day	2 feedings/day	3 feedings/day	>3 feedings/day	Do not know
Second milking of the dam	(8) ❑	(13) ❑	(14) ❑	(15) ❑	(5) ❑
Third milking of the dam	(8) ❑	(13) ❑	(14) ❑	(15) ❑	(5) ❑
Fourth or more milkings from dam	(8) ❑	(13) ❑	(14) ❑	(15) ❑	(5) ❑
Milk from other cows	(8) ❑	(13) ❑	(14) ❑	(15) ❑	(5) ❑

How many colostrum feedings do calves in <u>organic farms</u> receive from the:

	1 feeding/day	2 feedings/day	3 feedings/day	>3 feedings/day	Do not know
First milking of the dam	(8) ❑	(13) ❑	(14) ❑	(15) ❑	(5) ❑
Second milking of the dam	(8) ❑	(13) ❑	(14) ❑	(15) ❑	(5) ❑
Third milking of the dam	(8) ❑	(13) ❑	(14) ❑	(15) ❑	(5) ❑
Fourth or more milkings from dam	(8) ❑	(13) ❑	(14) ❑	(15) ❑	(5) ❑
Milk from other cows	(8) ❑	(13) ❑	(14) ❑	(15) ❑	(5) ❑

How many colostrum feedings do calves in biodynamic farms receive from the:

	1 feeding/day	2 feedings/day	3 feedings/day	>3 feedings/day	Do not know
First milking of the dam	(8) ❑	(13) ❑	(14) ❑	(15) ❑	(5) ❑
Second milking of the dam	(8) ❑	(13) ❑	(14) ❑	(15) ❑	(5) ❑
Third milking of the dam	(8) ❑	(13) ❑	(14) ❑	(15) ❑	(5) ❑
Fourth or more milkings from dam	(8) ❑	(13) ❑	(14) ❑	(15) ❑	(5) ❑
Milk from other cows	(8) ❑	(13) ❑	(14) ❑	(15) ❑	(5) ❑

CALVES – EARLY MILK FEEDING PERIOD (0-4 WEEKS OF AGE)

What is the most frequent way to milk-feed young calves after the separation from the cow?

	Open bucket or trough	Teat bucket or bar (teat feeder)	Automatic milk feeder via a teat	Foster (nurse) cow or dam (mother)	Other (specify below)	Do not know
Conventional herds	(1) ❑	(2) ❑	(3) ❑	(4) ❑	(5) ❑	(6) ❑
Organic herds	(1) ❑	(2) ❑	(3) ❑	(4) ❑	(5) ❑	(6) ❑
Biodynamic herds	(1) ❑	(2) ❑	(3) ❑	(4) ❑	(5) ❑	(6) ❑

How many litres of milk/milk replacer are fed to the calves at:

1st week of age

	<6 L/day	6-8 L/day	8-10 L/day	≥10 L/day	Do not know
Conventional farms	(8) ❑	(10) ❑	(11) ❑	(12) ❑	(5) ❑
Organic farms	(8) ❑	(10) ❑	(11) ❑	(12) ❑	(5) ❑
Biodynamic and others	(8) ❑	(10) ❑	(11) ❑	(12) ❑	(5) ❑

2nd week of age

	<6 L/day	6-8 L/day	8-10 L/day	≥10 L/day	Do not know
Conventional farms	(8) ❑	(10) ❑	(11) ❑	(12) ❑	(5) ❑
Organic farms	(8) ❑	(10) ❑	(11) ❑	(12) ❑	(5) ❑
Biodynamic and others	(8) ❑	(10) ❑	(11) ❑	(12) ❑	(5) ❑

3rd week of age

	<6 L/day	6-8 L/day	8-10 L/day	≥10 L/day	Do not know
Conventional farms	(8) ❑	(10) ❑	(11) ❑	(12) ❑	(5) ❑
Organic farms	(8) ❑	(10) ❑	(11) ❑	(12) ❑	(5) ❑
Biodynamic and others	(8) ❑	(10) ❑	(11) ❑	(12) ❑	(5) ❑

4th week of age

	<6 L/day	6-8 L/day	8-10 L/day	≥10 L/day	Do not know
Conventional farms	(8) ❑	(10) ❑	(11) ❑	(12) ❑	(5) ❑
Organic farms	(8) ❑	(10) ❑	(11) ❑	(12) ❑	(5) ❑
Biodynamic and others	(8) ❑	(10) ❑	(11) ❑	(12) ❑	(5) ❑

How many numbers of milk feedings are typically administered to calves at:

1st week of age

	1 feeding/day	2 feedings/day	3 feedings/day	>3 feedings/day	Do not know
Conventional farms	(8) ❑	(13) ❑	(14) ❑	(15) ❑	(5) ❑
Organic farms	(8) ❑	(13) ❑	(14) ❑	(15) ❑	(5) ❑
Biodynamic and others	(8) ❑	(13) ❑	(14) ❑	(15) ❑	(5) ❑

2nd week of age

	1 feeding/day	2 feedings/day	3 feedings/day	>3 feedings/day	Do not know
Conventional farms	(8) ❑	(13) ❑	(14) ❑	(15) ❑	(5) ❑
Organic farms	(8) ❑	(13) ❑	(14) ❑	(15) ❑	(5) ❑
Biodynamic and others	(8) ❑	(13) ❑	(14) ❑	(15) ❑	(5) ❑

3rd week of age

	1 feeding/day	2 feedings/day	3 feedings/day	>3 feedings/day	Do not know
Conventional farms	(8) ❑	(13) ❑	(14) ❑	(15) ❑	(5) ❑
Organic farms	(8) ❑	(13) ❑	(14) ❑	(15) ❑	(5) ❑
Biodynamic and others	(8) ❑	(13) ❑	(14) ❑	(15) ❑	(5) ❑

4th week of age

	1 feeding/day	2 feedings/day	3 feedings/day	>3 feedings/day	Do not know
Conventional farms	(8) ❑	(13) ❑	(14) ❑	(15) ❑	(5) ❑
Organic farms	(8) ❑	(13) ❑	(14) ❑	(15) ❑	(5) ❑
Biodynamic and others	(8) ❑	(13) ❑	(14) ❑	(15) ❑	(5) ❑

Does one daily milk feeding occur in the young milk-fed calves, and if so, please state the percentage of herds using this practice?

	Yes, to a great extend (> 75 % of farms)	Yes, to a moderate extend (>50 % of farms)	Yes, to a minor extend (< 25 % of farms)	Do not know
Conventional farms	(1) ❑	(6) ❑	(3) ❑	(5) ❑
Organic farms	(1) ❑	(6) ❑	(3) ❑	(5) ❑
Biodynamic and others	(1) ❑	(6) ❑	(3) ❑	(5) ❑

What type of milk is primarily fed to calves within the first 4 weeks?

	Whole milk from foster cow	Whole milk and milk replacer	Whole milk and waste milk	Waste milk and milk replacer	Milk replacer	Acidified/Yoghurt milk	Other (specify below)
Conventional farms	(13) ❑	(6) ❑	(8) ❑	(7) ❑	(14) ❑	(15) ❑	(16) ❑ ____
Organic farms	(13) ❑	(6) ❑	(8) ❑	(7) ❑	(14) ❑	(15) ❑	(16) ❑ ____
Biodynamic farms	(13) ❑	(6) ❑	(8) ❑	(7) ❑	(14) ❑	(15) ❑	(16) ❑ ____

If fed with waste milk, where does this milk most often come from (multiple answers possible):

	Cows treated with antibiotics or other medication	Cows with high SCC	Risk cows (e.g. Johne´s disease)	Other (please specify below)	Do not know
Conventional farms	(10) ❑	(7) ❑	(8) ❑	(9) ❑ ____	(5) ❑
Organic farms	(10) ❑	(7) ❑	(8) ❑	(9) ❑ ____	(5) ❑
Biodynamic and others	(10) ❑	(7) ❑	(8) ❑	(9) ❑ ____	(5) ❑

Housing & Management– Early milk feeding period

What is the most frequent way to house calves for the first week(s) after being separated from the cow?

	Individual pen or hutch	Pair pen or hutch	Group pen or hutch	With foster cow or dam	Other (please specify below)	Do not know
Conventional farms	(3) ❑	(4) ❑	(5) ❑	(6) ❑	(7) ❑ ____	(8) ❑
Organic farms	(3) ❑	(4) ❑	(5) ❑	(6) ❑	(7) ❑ ____	(8) ❑
Biodynamic farms	(3) ❑	(4) ❑	(5) ❑	(6) ❑	(7) ❑ ____	(8) ❑

Are artificially milk fed calves required by legislation or codes of practice to have access to an artificial teat (e.g. teat bucket, teat feeder, teat bar, dummy teat)?

	No	Yes, by legislation (please specify below)	Yes, by codes of practice (please specify, e.g. insert link)	Do not know
Conventional farms	(1) ❑	(6) ❑ ____	(7) ❑ ____	(5) ❑
Organic farms	(1) ❑	(6) ❑ ____	(7) ❑ ____	(5) ❑
Biodynamic and others	(1) ❑	(6) ❑ ____	(7) ❑ ____	(5) ❑

What is the most frequent method of providing artificial teats?

	Permanent access (e.g. mounted artificial teat in box or automatic milk feeder)	Access at feeding with artificial teat (e.g. bucket removed after feeding)	No access	Do not know
Conventional herds	(5) ❑	(6) ❑	(7) ❑	(8) ❑
Organic herds	(5) ❑	(6) ❑	(7) ❑	(8) ❑
Biodynamic herds	(5) ❑	(6) ❑	(7) ❑	(8) ❑

Do farms provide artificial teats to the milk-fed calves?

	Yes, to a great extend (> 75 % of farms)	Yes, to a moderate extend (>50 % of farms)	Yes, to a minor extend (< 25 % of farms)	Do not know
Conventional farms	(1) ❑	(6) ❑	(3) ❑	(5) ❑
Organic farms	(1) ❑	(6) ❑	(3) ❑	(5) ❑
Biodynamic and others	(1) ❑	(6) ❑	(3) ❑	(5) ❑

Are there any legislative requirements or codes of practice for housing calves under 8 weeks?

	No	Yes, by legislation (please specify below)	Yes, by codes of practice (please specify, e.g. insert link)	Do not know
Conventional farms	(1) ❑	(6) ❑ ____	(7) ❑ ____	(5) ❑
Organic farms	(1) ❑	(6) ❑ ____	(7) ❑ ____	(5) ❑
Biodynamic and others	(1) ❑	(6) ❑ ____	(7) ❑ ____	(5) ❑

CALVES – LATE MILK FEEDING PERIOD (5-8 WEEKS OF AGE)

What is the most frequent way to milk-feed calves at 5-8 weeks of age?

	Open bucket or through	Teat bucket or bar (teat feeder)	Automatic feeder via a teat	Foster (nurse) cow or dam (mother)	Other (specify below)	Do not know
Conventional farms	(13) ❑	(6) ❑	(14) ❑	(7) ❑	(15) ❑ ____	(16) ❑
Organic farms	(13) ❑	(6) ❑	(14) ❑	(7) ❑	(15) ❑ ____	(16) ❑
Biodynamic farms	(13) ❑	(6) ❑	(14) ❑	(7) ❑	(15) ❑ ____	(16) ❑

How many litres of milk/milk replacer are fed to the calves at:

5th week of age

	<6 L/day	6-8 L/day	8-10 L/day	≥10 L/day	Do not know
Conventional farms	(8) ❑	(10) ❑	(11) ❑	(12) ❑	(5) ❑
Organic farms	(8) ❑	(10) ❑	(11) ❑	(12) ❑	(5) ❑
Biodynamic and others	(8) ❑	(10) ❑	(11) ❑	(12) ❑	(5) ❑

6th week of age

	<6 L/day	6-8 L/day	8-10 L/day	≥10 L/day	Do not know
Conventional farms	(8) ❑	(10) ❑	(11) ❑	(12) ❑	(5) ❑
Organic farms	(8) ❑	(10) ❑	(11) ❑	(12) ❑	(5) ❑
Biodynamic and others	(8) ❑	(10) ❑	(11) ❑	(12) ❑	(5) ❑

7th week of age

	<6 L/day	6-8 L/day	8-10 L/day	≥10 L/day	Do not know
Conventional farms	(8) ❑	(10) ❑	(11) ❑	(12) ❑	(5) ❑
Organic farms	(8) ❑	(10) ❑	(11) ❑	(12) ❑	(5) ❑
Biodynamic and others	(8) ❑	(10) ❑	(11) ❑	(12) ❑	(5) ❑

8th week of age

	<6 L/day	6-8 L/day	8-10 L/day	≥10 L/day	Do not know
Conventional farms	(8) ❑	(10) ❑	(11) ❑	(12) ❑	(5) ❑
Organic farms	(8) ❑	(10) ❑	(11) ❑	(12) ❑	(5) ❑
Biodynamic and others	(8) ❑	(10) ❑	(11) ❑	(12) ❑	(5) ❑

How many numbers of milk feedings are typically administered to calves at:

5th week of age

	1 feeding/day	2 feedings/day	3 feedings/day	>3 feedings/day	Do not know
Conventional farms	(8) ❑	(13) ❑	(14) ❑	(15) ❑	(5) ❑
Organic farms	(8) ❑	(13) ❑	(14) ❑	(15) ❑	(5) ❑
Biodynamic and others	(8) ❑	(13) ❑	(14) ❑	(15) ❑	(5) ❑

6th week of age

	1 feeding/day	2 feedings/day	3 feedings/day	>3 feedings/day	Do not know
Conventional farms	(8) ❑	(13) ❑	(14) ❑	(15) ❑	(5) ❑
Organic farms	(8) ❑	(13) ❑	(14) ❑	(15) ❑	(5) ❑
Biodynamic and others	(8) ❑	(13) ❑	(14) ❑	(15) ❑	(5) ❑

7th week of age

	1 feeding/day	2 feedings/day	3 feedings/day	>3 feedings/day	Do not know
Conventional farms	(8) ❑	(13) ❑	(14) ❑	(15) ❑	(5) ❑
Organic farms	(8) ❑	(13) ❑	(14) ❑	(15) ❑	(5) ❑
Biodynamic and others	(8) ❑	(13) ❑	(14) ❑	(15) ❑	(5) ❑

8th week of age

	1 feeding/day	2 feedings/day	3 feedings/day	>3 feedings/day	Do not know
Conventional farms	(8) ❑	(13) ❑	(14) ❑	(15) ❑	(5) ❑
Organic farms	(8) ❑	(13) ❑	(14) ❑	(15) ❑	(5) ❑
Biodynamic and others	(8) ❑	(13) ❑	(14) ❑	(15) ❑	(5) ❑

Does one daily milk feeding occur in the older milk-fed calves, and if so, please estimate the percentage of herds using this practice?

Conventional herds _____

Organic herds _____

Biodynamic herds _____

Not applicable (please set at 100) _____

If one daily feeding is used, at what age do farms usually start this practice?
(Please enter age in weeks)

Conventional herds

Organic herds _____

Biodynamic herds	_____
Not applicable (please set at 100)	_____

Are older calves milk-fed ad libitum?

	Yes, to a great extend (> 75 % of farms)	Yes, to a moderate extend (>50 % of farms)	Yes, to a minor extend (< 25 % of farms)	Do not know
Conventional farms	(1) ❑	(6) ❑	(3) ❑	(5) ❑
Organic farms	(1) ❑	(6) ❑	(3) ❑	(5) ❑
Biodynamic and others	(1) ❑	(6) ❑	(3) ❑	(5) ❑

What type of milk is primarily fed to calves between 5- 8 weeks of age?

	Whole milk from foster cow	Whole milk and milk replacer	Whole milk and waste milk	Waste milk	Waste milk and milk replacer	Milk replacer	Acidified/ Yoghurt milk	Other (please specify below)
Conventional farms	(13) ❑	(6) ❑	(7) ❑	(8) ❑	(9) ❑	(10) ❑	(11) ❑	(12) ❑ _____
Organic farms	(13) ❑	(6) ❑	(7) ❑	(8) ❑	(9) ❑	(10) ❑	(11) ❑	(12) ❑ _____
Biodynamic farms	(13) ❑	(6) ❑	(7) ❑	(8) ❑	(9) ❑	(10) ❑	(11) ❑	(12) ❑ _____

If fed with waste milk, where does this milk come from:

	Cows treated with antibiotics or other medication	Cows with high SCC	Risk cows (e.g. Johne´s disease)	Other (please specify below)	Do not know
Conventional farms	(10) ❑	(7) ❑	(8) ❑	(9) ❑ ____	(5) ❑
Organic farms	(10) ❑	(7) ❑	(8) ❑	(9) ❑ ____	(5) ❑
Biodynamic and others	(10) ❑	(7) ❑	(8) ❑	(9) ❑ ____	(5) ❑

Housing – Late milk feeding period

What is the most frequent way to house calves calves at age 5-8 weeks?

	Individual pen or hutch	Pair pen or hutch	Group pen or hutch	With foster cow or dam	Other (please specify below)	Do not know
Conventional farms	(3) ❑	(4) ❑	(5) ❑	(6) ❑	(7) ❑ ____	(8) ❑
Organic farms	(3) ❑	(4) ❑	(5) ❑	(6) ❑	(7) ❑ ____	(8) ❑
Biodynamic farms	(3) ❑	(4) ❑	(5) ❑	(6) ❑	(7) ❑ ____	(8) ❑

Weaning of milk

Which factors determine weaning of calves?

Please rank the factors from the most frequent to the least frequent

Weaning in conventional farms

	1	2	3	4	5	6
Age	(1) ❑	(2) ❑	(3) ❑	(4) ❑	(5) ❑	(6) ❑
Size	(1) ❑	(2) ❑	(3) ❑	(4) ❑	(5) ❑	(6) ❑
Weight	(1) ❑	(2) ❑	(3) ❑	(4) ❑	(5) ❑	(6) ❑
Concentrate intake	(1) ❑	(2) ❑	(3) ❑	(4) ❑	(5) ❑	(6) ❑
Other (specify below in the comments box)	(1) ❑	(2) ❑	(3) ❑	(4) ❑	(5) ❑	(6) ❑
Do not know	(1) ❑	(2) ❑	(3) ❑	(4) ❑	(5) ❑	(6) ❑

Weaning in organic farms

	1	2	3	4	5	6
Age	(1) ❑	(2) ❑	(3) ❑	(4) ❑	(5) ❑	(6) ❑
Size	(1) ❑	(2) ❑	(3) ❑	(4) ❑	(5) ❑	(6) ❑
Weight	(1) ❑	(2) ❑	(3) ❑	(4) ❑	(5) ❑	(6) ❑
Concentrate intake	(1) ❑	(2) ❑	(3) ❑	(4) ❑	(5) ❑	(6) ❑

	1	2	3	4	5	6
Other (specify below in the comments box)	(1) ❑	(2) ❑	(3) ❑	(4) ❑	(5) ❑	(6) ❑
Do not know	(1) ❑	(2) ❑	(3) ❑	(4) ❑	(5) ❑	(6) ❑

Weaning in biodynamic farms

	1	2	3	4	5	6
Age	(1) ❑	(2) ❑	(3) ❑	(4) ❑	(5) ❑	(6) ❑
Size	(1) ❑	(2) ❑	(3) ❑	(4) ❑	(5) ❑	(6) ❑
Weight	(1) ❑	(2) ❑	(3) ❑	(4) ❑	(5) ❑	(6) ❑
Concentrate intake	(1) ❑	(2) ❑	(3) ❑	(4) ❑	(5) ❑	(6) ❑
Other (specify below in the comments box)	(1) ❑	(2) ❑	(3) ❑	(4) ❑	(5) ❑	(6) ❑
Do not know	(1) ❑	(2) ❑	(3) ❑	(4) ❑	(5) ❑	(6) ❑

At what age is weaning completed?

	6-8 weeks	8-10 weeks	≥10 weeks	Do not know
Conventional farms	(13) ❑	(14) ❑	(15) ❑	(5) ❑
Organic farms	(13) ❑	(14) ❑	(15) ❑	(5) ❑
Biodynamic and others	(13) ❑	(14) ❑	(15) ❑	(5) ❑

What is the most frequently applied weaning practice?

Please rank the factors from the most frequent to the least frequent

Conventional farms

Gradual weaning with declining milk volume over more than 7 days

Gradual weaning with declining milk volume over 5-7 days

Gradual weaning with declining milk volume over less than 5 days

Abrupt weaning

Other (specify below in the comments box)

Organic farms

Gradual weaning with declining milk volume over more than 7 days

Gradual weaning with declining milk volume over 5-7 days

Gradual weaning with declining milk volume over less than 5 days

Abrupt weaning

Other (specify below in the comments box)

Biodynamic farms

Gradual weaning with declining milk volume over more than 7 days

Gradual weaning with declining milk volume over 5-7 days

Gradual weaning with declining milk volume over less than 5 days

Abrupt weaning

Other (specify below in the comments box)

Storing information requires your consent. Before storage and analysis, the data will be anonymized

(1) ❑ I agree that my reply to this questionnaire can be stored in anonymized form by the University of Copenhagen.

We kindly thank you for your answers and valuable time.
